# Draft Genome Sequence of MPKL 26, the Type Strain of the Novel Species *Sinomonas mesophila*

**DOI:** 10.1128/genomeA.00247-17

**Published:** 2017-04-27

**Authors:** Manik Prabhu Narsing Rao, Jian-Yu Jiao, Lan Liu, Bao-Zhu Fang, Xiao-Tong Zhang, Wei Chen, Jiao Zhao, Min Xiao, Wen-Jun Li

**Affiliations:** a State Key Laboratory of Biocontrol and Guangdong Key Laboratory of Plant Resources, School of Life Sciences, Sun Yat-Sen University, Guangzhou, PR China; b China Tobacco Yunnan Industrial Co. Ltd., Kunming, PR China; c Beijing Genomics Institute at Shenzhen, Shenzhen, PR China; d Key Laboratory of Biogeography and Bioresources in Arid Land, Xinjiang Institute of Ecology and Geography, Chinese Academy of Sciences, Ürmqi, PR China

## Abstract

*Sinomonas mesophila* MPKL 26^T^ can produce silver nanoparticles. Here, we present the 4.0-Mb genome of this type strain, which contains 47 scaffolds with an *N*_50_ scaffold length of 261,266 bp. The availability of the genome sequence will provide a better understanding of strain MPKL 26^T ^and the genus *Sinomonas*.

## GENOME ANNOUNCEMENT

The genus *Sinomonas* was proposed by Zhou et al. (1) and comprises 10 species of Gram-positive actinobacteria (http://www.bacterio.net/sinomonas.html [2]). Members of the genus *Sinomonas* have the ability to produce silver nanoparticles (3), can hydrolyze starch (4), and are used for biodesulfurization coal (5). Here, we report the draft genome sequence of strain MPKL 26, the type strain of the novel species *Sinomonas mesophila*, which has the ability to produce silver nanoparticles (3).

Whole-genome sequencing of *S. mesophila* strain MPKL 26^T^ was performed using a paired-end sequencing method with the HiSeq 2000 platform (Illumina, San Diego, CA, USA). Using 1.49 Gb of clean data, the draft genome was assembled with the SOAPdenovo2 package (6). The draft genome of *S. mesophila* MPKL 26^T^ contains 47 scaffolds with a total size of 4,008,197 bp and with an *N*_50_ scaffold length of 261,266 bp. The G+C content is 71.1%. Gene prediction was carried out using Glimmer version 3.02 (7). The identification of tRNAs and rRNAs was carried out using tRNAscan-SE version 1.23 (8) and RNAmmer version 1.2 (9). A total of 3,742 genes were predicted with four rRNA and 52 tRNA genes. The predicted coding sequences were translated and used to search against the KEGG and COG databases. The functional annotation of the genome was also performed with RAST (10), which revealed 448 genes for carbohydrate metabolism, 235 genes involved in protein metabolism, and 90 genes involved in DNA metabolism. These data sources were combined to assert a product description for each predicted protein.

### Accession number(s).

This whole-genome shotgun project has been deposited in DDBJ/EMBL/GenBank under the accession number MTIC00000000. The version described in this paper is the first version, MTIC01000000.
